# Strategies to mitigate emergency department overcrowding: enhancing healthcare efficiency and patient care

**DOI:** 10.17179/excli2024-8044

**Published:** 2025-01-23

**Authors:** Mahmoudreza Peyravi, Mohammad Ebrahimi, Marziyeh Hamyali Ainvand

**Affiliations:** 1 Department of Health in Disasters and Emergencies, School of Health Management and Information Sciences, Shiraz University of Medical Sciences, Shiraz, Iran; 2 Student Research Committee, Shiraz University of Medical Sciences, Shiraz, Iran

## ⁯

Excessive overcrowding in emergency departments (ED) has become a global challenge, leading to decreased quality of healthcare services, delayed patient treatment, and increased mortality rates. This issue is particularly pronounced in developing countries due to the inefficiency of healthcare systems and a lack of resources (Di Somma et al., 2015[2]; Haraldsson et al., 2024[4]; Larsen et al., 2024[5]; Nan et al., 2024[6]). Overcrowding in emergency departments (ED) is driven by several factors, including population growth, an aging demographic, inadequate hospital resources, the increasing complexity of patient needs, and operational challenges faced by healthcare providers (Haraldsson et al., 2024[4]; Bütün et al., 2024[1]; Haas et al., 2023[3]; Rader and Ritchie, 2023[7]).

Given the importance of overcrowding in emergency departments (ED), this study proposes strategies to address this issue. Implementing the recommended measures could improve healthcare quality, reduce costs, and enhance patient satisfaction. 

### Strategies for reducing overcrowding in hospital emergency departments (ED):

*Establishing hospice centers and home care services:* Developing specialized facilities for end-of-life patients can alleviate pressure on emergency departments. These centers play a critical role in symptom management, reducing costs, and enhancing the quality of life for both patients and their families.

*Restricting elective Surgeries in Emergency Operating Rooms:* Performing non-urgent surgeries in emergency spaces leads to unnecessary resource utilization and delays in treating emergency patients. By establishing clear protocols and training healthcare staff, resources can be optimized effectively.

*Hospital bed management:* Implementing flexible bed management systems, such as digital dashboards and Internet of Things (IoT) technologies, can reduce patient wait times and improve service quality. 

*Enhancing access to medical equipment:* Ensuring adequate availability of equipment across hospital departments can help alleviate the burden on emergency departments. Investment in resources and continuous staff training are essential for achieving this goal.

*Expanding general hospitals:* To reduce the strain on public hospital emergency departments, the development of general hospitals and the establishment of effective referral systems are essential. Additionally, expanding outpatient clinics can help decrease unnecessary visits to emergency departments.

*Admitting patients to private hospital emergency departments:* Encouraging private hospitals to expand their emergency services and accept additional patients can help ease the burden on general hospitals.

*Reducing unnecessary referrals to the emergency department:* Referrals caused by equipment shortages or diagnostic errors should be minimized by strengthening infrastructure, training healthcare staff, and raising public awareness.

*Utilizing increased surge capacity:* Creating temporary beds during critical situations and expanding outpatient services can help reduce wait times and workload pressure. Additionally, improving the management of existing spaces and utilizing patient management technologies to decrease unnecessary visits can be effective.

*Role of senior faculty in emergency management:* The presence of experienced faculty members during emergency rounds, their ability to make quick decisions regarding patient care, and managing referrals can enhance system efficiency and improve the quality of care.

*Integration of prehospital and hospital management:* Enhanced coordination between these two sectors, especially in developing countries, can facilitate patient transfer processes and reduce pressure on emergency departments.

*Control of admissions from other areas:* Assigning a senior faculty member to manage patient admissions from other regions and oversee the process helps reduce unnecessary cases. Continuous analysis of admission data provides valuable insights for improving management.

*Management of discharge delays:* Delays in tests, patient discharge, and administrative processes contribute to increased emergency department congestion and reduced efficiency. Regular monitoring and data analysis help identify issues and implement corrective measures. Timely management of these processes can increase hospital capacity and reduce congestion.

*Conducting mortality review committees:* Regular meetings to analyze the causes of patient mortality, revise procedures, and reduce clinical errors are essential. The outcomes of these sessions must be effectively followed up to ensure improvements are made.

Emergency department (ED) overcrowding is a significant challenge faced by healthcare systems worldwide. It adversely affects the quality of care, patient outcomes, and the efficiency of healthcare providers. Solutions such as establishing alternative care centers, improving infrastructure, optimizing bed capacity, and strengthening referral systems can play a crucial role in alleviating this crisis. Implementing these strategies will not only enhance the quality of services but also reduce costs, improve patient satisfaction, and increase the overall efficiency of the healthcare system. These measures can be adapted and customized to suit local conditions in any country. Continuous evaluation and updating of these approaches are essential for their long-term sustainability and effectiveness.

## Conflict of interest

None to declare.
